# Intravascular T-cell lymphoma in a patas monkey (*Erythrocebus patas*)

**DOI:** 10.5194/pb-4-39-2017

**Published:** 2017-03-08

**Authors:** Karen Lampe, Jens-Christian Rudnick, Fabian Leendertz, Martina Bleyer, Kerstin Mätz-Rensing

**Affiliations:** 1 Pathology Unit, German Primate Center, Leibniz-Institute for Primate Research, Kellnerweg 4, 37077 Göttingen, Germany; 2 Tierklinik-Tierheim GmbH Rostock, Thierfelderstraße 19, 18059 Rostock, Germany; 3 Robert Koch Institut, Nordufer 20, 13353 Berlin, Germany

## Abstract

A 9-year-old female captive patas monkey (*Erythrocebus patas*) presented with poor general condition, inability to stand, petechiae, anaemia, thrombocytopenia, and leukocytosis. Due to poor response to treatment, the animal was euthanized 16 days later. Postmortem examination revealed hemorrhages in several organs and bilateral cerebral infarctions. Histologically, prominent accumulations of large neoplastic lymphocytes in cerebral and meningeal blood vessels were demonstrated within the lesions and in other organs (e.g., bone marrow, ovary, intestine). Immunohistochemically, neoplastic cells expressed CD3 and Ki-67. PCR revealed a lymphocryptovirus (LCV) infection, while Epstein–Barr nuclear antigen 2 (EBNA2) could not be demonstrated within neoplastic cells by means of immunohistochemistry. Based on the pathological findings, an intravascular lymphoma (IVL) of T-cell origin was diagnosed. To the authors' knowledge, this is the first report on this rare entity in a nonhuman primate.

## 1 Introduction

Intravascular lymphoma (IVL) is a rare type of non-Hodgkin's lymphoma which is characterized by proliferation of neoplastic lymphocytes confined to blood vessel lumina in the absence of a primary extravascular tumor mass (Zuckerman et al., 2006; Ponzoni et al., 2007). In 1959, the entity was first described by Pfleger and Tappeiner as “angioendotheliomatosis proliferans systemisata”, referring to its suspected endothelial cell origin (Pfleger and Tappeiner, 1959). However, immunohistochemical investigations in the middle of the 1980s revealed the lymphocytic phenotype of the neoplastic cells, giving rise to reclassification of the neoplasm as “angiotropic large cell lymphoma” and “intravascular lymphomatosis” (Sheibani et al., 1986; Wick et al., 1986; Ferry et al., 1988). Apart from humans, IVL has been reported in a range of domestic animals, including dogs (Cullen et al., 2000; McDonough et al., 2002; Lane et al., 2012), cats (Lapointe et al., 1997; Henrich et al., 2007), and a horse (Raidal et al., 2006). Since the majority of cases in people are of B-cell origin with rare cases of T-cell and natural killer (NK)-cell tumors (Wick and Mills, 1991; Estalilla et al., 1999; Ferreri et al., 2004; Ponzoni and Ferreri, 2006; Zuckerman et al., 2006), only the B-cell IVL is listed in the World Health Organization classification of hematopoietic tumors, defining it as a rare variant of extranodal large B-cell lymphoma with selective intravascular growth (Nakamura et al., 2008). Cases in animals predominantly display a T-cell or a non-T-cell, non-B-cell phenotype (McDonough et al., 2002; Raidal et al., 2006). The clinical presentation of this systemic disease is diverse and depends on the spectrum of affected organs, rendering ante mortem diagnosis difficult (Ferreri et al., 2004; Zuckerman et al., 2006). Progressive occlusion of small vessels by neoplastic cells may result in thrombosis, hemorrhage, and infarction (Cullen et al., 2000; Bush et al., 2003). In humans, there is a clear relationship between T-cell IVL and Epstein–Barr virus (EBV, human herpes virus 4) infection, as demonstrated by detection of EBV RNA in lymphoma cells (Au et al., 1997; Cerroni et al., 2008).

In Old World monkeys, lymphomas are naturally occurring neoplasms. The vast majority of them are associated with certain viral agents (Bruce et al., 2012; Hirata et al., 2015; Hubbard et al., 1993; Hunt et al., 1983; Miller, 2012; Paramastri et al., 2002; Suzuki et al., 2005), whose natural host spectrum covers different African and Asian nonhuman primate species (Carville and Mansfield, 2008; Lerche and Osborn, 2003). In macaques, these agents include simian retroviruses, in particular simian immunodeficiency virus (SIV; Habis et al., 1999; Mätz-Rensing et al., 1999; Rivailler et al., 2004) and simian retrovirus (SRV) type D (Paramastri et al., 2002). In addition to the retrovirus-induced immunodeficiency, the development of lymphomas is thought to be associated with coinfection with one of two types of gammaherpesviruses, namely simian lymphocryptoviruses (LCVs), the simian equivalent of EBV (Blaschke et al., 2001; Bruce et al., 2012; Carville and Mansfield, 2008; Habis et al., 2000; Kahnt et al., 2002; Li et al., 1993; Mätz-Rensing et al., 1999; Pingel et al., 1997), and rhadinoviruses (Bruce et al., 2012; Orzechowska et al., 2008). In contrast to the aforementioned retroviruses, simian T-cell lymphotropic virus (STLV) associated lymphomagenesis in nonhuman primates does not seem to require a herpesviral co-infection (Allan et al., 2001; Homma et al., 1984; Hubbard et al., 1993).

In the present case report, we describe the clinical, morphological, and immunophenotypical features of an IVL in a captive patas monkey (*Erythrocebus patas*).

## 2 Case report

### 2.1 Clinical presentation

A 9-year-old female captive patas monkey (*Erythrocebus patas*), born and raised in Rostock Zoo, Rostock, Germany, was found in lateral recumbency with poor general condition. The animal was part of a breeding group of three male and three female patas monkeys kept in a combined indoor and outdoor caging. In the same building, putty-nosed monkeys (*Cercopithecus nictitans*) and lion-tailed macaques (*Macaca silenus*) were housed. Due to suspected yersiniosis, treatment of the patas monkey was initiated, including administration of Synulox (Zoetis Deutschland GmbH, Berlin, Germany, 25 mg kg day${}^{-1}$ s.c.), followed by doxycycline (ratiopharm GmbH, Ulm, Germany, 20 mg twice daily p.o.) and prednisolone (ratiopharm GmbH, Ulm, Germany, 5 mg twice daily p.o.), as well as intravenous fluid therapy (Hartmann/Ringer's lactate solution, B. Braun VetCare GmbH, Melsungen, Germany, 10 mL kg${}^{-1}$ i.v.). Treatment resulted in a slight improvement of the general condition. However, the animal still showed weakness, staggering, anorexia, petechiae, and ecchymoses in the skin and oral mucosa. Hematological analysis revealed mild anaemia (erythrocyte count: 4.07 T L${}^{-1}$, reference range 5.05–5.99 T L${}^{-1}$; hematocrit (Hct): 0.27 L L${}^{-1}$, reference range 0.41–0.47 L L${}^{-1}$; hemoglobin (Hb): 5,23 mmol L${}^{-1}$, reference range 8.38–9.62 mmol L${}^{-1}$; mean corpuscular volume (MCV): 66 fl, reference range 74.3–85.9 fl; mean corpuscular hemoglobin (MCH): 1.28 fmol, reference range 1.5–1.75 fmol; mean corpuscular hemoglobin concentration (MCHC): 19.38 mmol L${}^{-1}$, reference range 19.9–20.9 mmol L${}^{-1}$) and severe thrombocytopenia (platelet count 48 G L${}^{-1}$, reference range 212–380 G L${}^{-1}$), as well as marked leukocytosis (white blood cells (Wbc): 9.66 G L${}^{-1}$, reference range 3.7–6.9 G L${}^{-1}$; reference ranges adapted from Sly et al., 1978). PCR on a blood sample targeting *Babesia*, *Ehrlichia*, and *Anaplasma* yielded negative results. Due to a deteriorating condition with poor response to treatment, the monkey was euthanized 16 days after onset of clinical signs and submitted to the Pathology Unit of the German Primate Center.

### 2.2 Materials and methods

Postmortem examination was performed according to a standard necropsy protocol. Representative tissue samples were taken, fixed in 10 % neutral buffered formalin, processed routinely, and embedded in paraffin wax. Subsequently, 4 µm sections were mounted on glass slides and stained with hematoxylin and eosin (H & E). Immunohistochemical stains (IHC; streptavidin–biotin peroxidase (SABC) method) were performed on formalin-fixed paraffin wax-embedded tissue using a panel of primary antibodies, including rabbit anti-human CD3 (clone T3-4 B5, dilution 1 in 50; Dako, Glostrup, Denmark), mouse anti-human CD20 (clone L26, dilution 1 in 300; Dako), mouse anti-human CD68 (clone KP1, dilution 1 in 100; Dako), mouse anti-human Ki-67 (clone MIB1, dilution 1 in 50; Dako), mouse anti-human CD4 (clone 1F6, dilution 1 in 20; Leica, Newcastle, UK), mouse anti-human CD8 (clone SP57, ready to use; Ventana, Tucson, USA), and mouse anti-EBNA2 (Epstein–Barr nuclear antigen 2; dilution 1 in 10; abcam, Cambridge, UK). All antibodies were monoclonal and required epitope retrieval by means of heat and, except for the anti-EBNA2 antibody, EDTA pretreatment. For each protocol, appropriate positive and negative controls were used.

Tissue specimens from the brain were tested for a panel of viral agents known to be related to lymphomagenesis in monkeys by means of PCR. DNA was extracted using the DNA tissue kit (Qiagen, Hilden, Germany), and PCRs were performed for the detection of SIV (Clewley et al., 1998), simian T-cell leukemia virus type 1 (STLV-1; Leendertz et al., 2010), and herpes viruses, using pan-herpes primers (Chmielewicz et al., 2003). PCR products were purified using the Qiaquick PCR purification kit (Qiagen) and sequenced directly in both directions without interim cloning.

## 3 Results

### 3.1 Macroscopic findings

**Figure 1:**
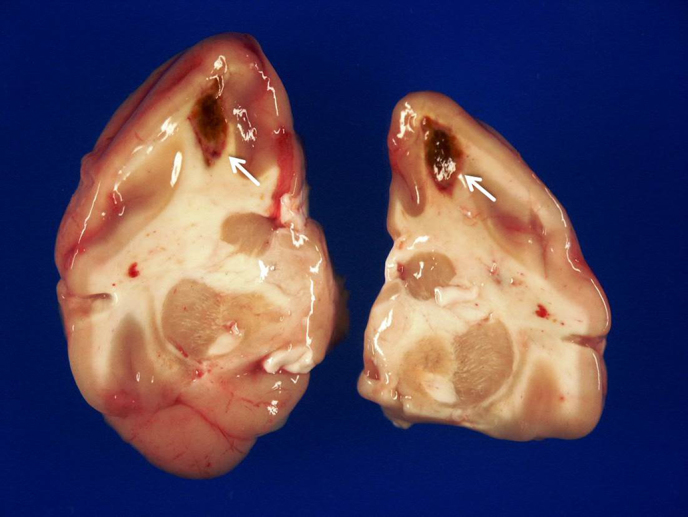
Infarctions with hemorrhages (white arrows) in both cerebral hemispheres.

Upon gross examination, oligofocal red to dark brown areas ranging between 3 and 7 mm in diameter, consistent with infarctions, were detected in both cerebral hemispheres (Fig. 1). Moreover, multifocal petechial to ecchymotic hemorrhages were present in various organs (e.g., subcutis of the trunk, uterus, and urinary bladder). There was a mild serosanguinous pericardial effusion and mild splenomegaly. Inguinal and axillary lymph nodes were moderately enlarged, whereas visceral lymph nodes appeared normal.

**Figure 2:**
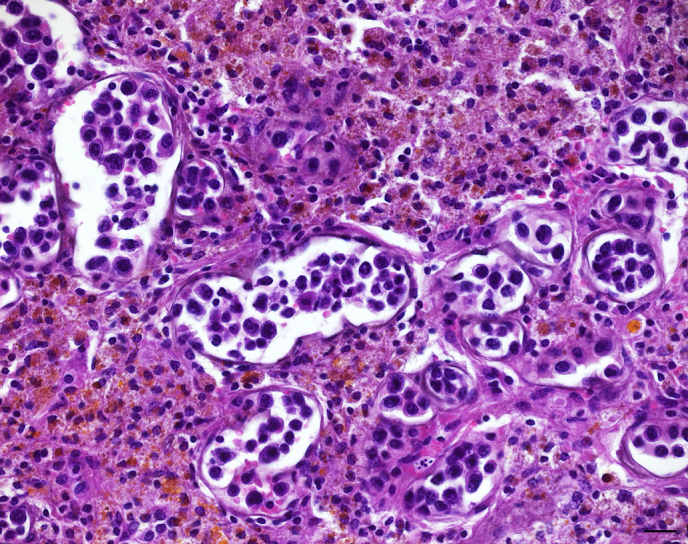
Cerebral capillaries distended by intravascular emboli of large pleomorphic neoplastic lymphocytes. Adjacent cerebral parenchyma displays loss of architecture, infiltration with numerous foamy macrophages, lymphocytes, as well as hemosiderin and hematoidin deposition. H & E. Bar: 20 µm.

### 3.2 Histologic and immunohistologic findings

**Figure 3:**
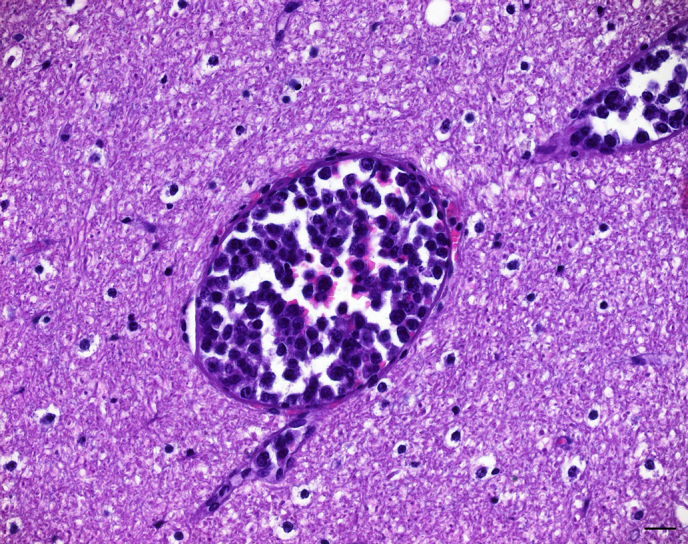
Corresponding emboli of neoplastic lymphocytes distending multiple capillaries within the cerebellum. The adjacent cerebellar parenchyma appears normal. H & E. Bar: 20 µm.

**Figure 4:**
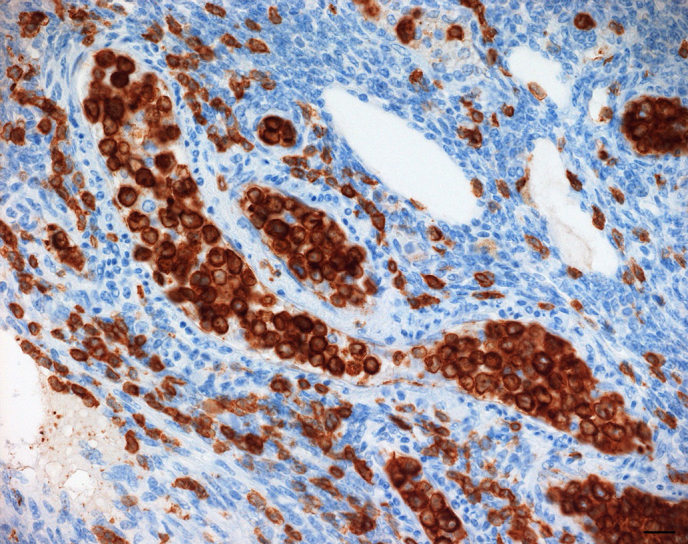
Accumulation of large neoplastic lymphocytes filling the lumina of multiple venules and capillaries within the ovary. Neoplastic cells show a strong cytoplasmic CD3 expression. IHC, SABC method. Bar: 20 µm.

**Figure 5:**
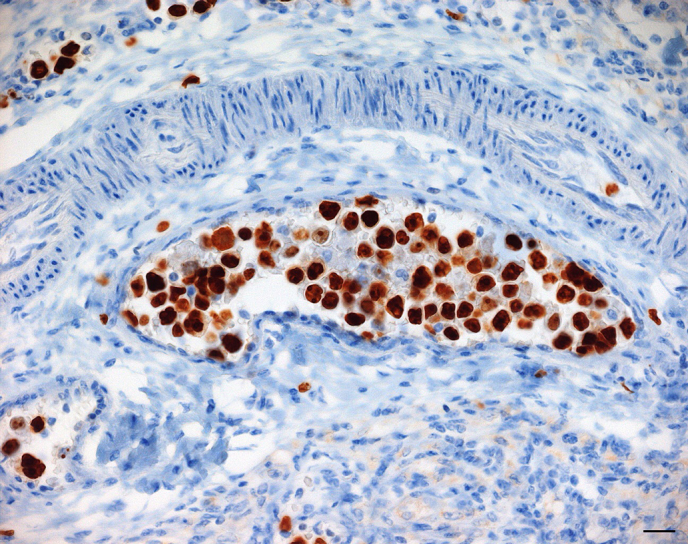
Venous vessel of a mesenterial lymph node with intravascular neoplastic lymphocytes highlighted by a marked nuclear Ki-67 signal. IHC, SABC method. Bar: 20 µm.

Histological examination revealed a lymphoproliferative process with a striking restriction to blood vessel lumina. In the areas of cerebral infarction (Fig. 2) as well as within meninges, prominent multifocal emboli of large neoplastic lymphocytes within capillaries and small caliber venous vessels were observed. Occasionally, affected vessels were markedly distended by neoplastic cells (Fig. 2). The latter were characterized by moderate anisocytosis and anisokaryosis showing moderate amounts of homogenous to finely vacuolated amphophilic cytoplasm with distinct cellular borders, irregularly round to polygonal nuclei with coarsely clumped chromatin, and one to several variably distinct nucleoli. Mitotic figures averaged four per high power field, occasionally displaying a bizarre appearance. Upon careful screening, a corresponding intravascular neoplastic cell population was found in a range of additional organs including cerebellum (Fig. 3), spleen, mesenterial lymph nodes, bone marrow sinusoids, ovary, haired skin, and intestine. Infrequently, infiltration of the adjacent parenchyma by few neoplastic cells was observed within the cerebrum and cerebellum, whereas no extravascular neoplastic mass could be detected. While some of the occluded vessels within the cerebrum were associated with extensive loss of architecture, hemorrhage, hemosiderin and hematoidin deposition, as well as moderate histiocytic and lymphocytic infiltration of the adjacent cerebral parenchyma, no secondary lesions were observed in the other affected organs. In the liver and in the kidneys, a low-grade to moderate multifocal inflammatory infiltrate was present, consisting of small mature lymphocytes, and affecting hepatic sinusoids and portal triads as well as renal interstitium respectively. The axillary and inguinal lymph nodes displayed marked paracortical and low-grade to moderate follicular hyperplasia. In the bone marrow moderate nodular lymphoid hyperplasia was present. There was hematopoietic activity in all three lineages. Immunohistochemically, the intravascular neoplastic cell population virtually uniformly expressed CD3, characterized by a strong cytoplasmic signal (Fig. 4), while it was negative for CD20, and CD68. There was no evidence for expression of CD4 and CD8 in either tumor cells or normal lymphoid tissue. Furthermore, 80–90 % of the neoplastic cells showed a strong nuclear Ki-67 signal (Fig. 5). The hyperplastic lymphoid aggregates in the bone marrow were predominantly composed of CD3+ T-cells, surrounded by a rim of CD20+ B-cells. Neoplastic cells showed no immunoreactivity for the EBV-marker EBNA2.

### 3.3 Results of molecular analyses

PCR amplification of tumor-bearing brain tissue yielded a strong signal for lymphocryptovirus. The sequences obtained were closely related to a previously published LCV-1 in a patas monkey (Ehlers et al., 2003). SIV and STLV-1 were not detected in tumor-bearing tissues by means of PCR.

## 4 Discussion

Given the striking confinement of large neoplastic lymphocytes to vascular lumina without detection of an extravascular tumor mass and the uniform CD3 expression by neoplastic cells, an IVL of T-cell phenotype was diagnosed. In contrast to the present case, lymphomas in species closely related to the patas monkey, such as macaques and baboons, show a completely different morphology. They present as lymph node enlargements or distinct masses of viscera or diffuse infiltrations of organs and thus do not exclusively affect the vascular system (Bruce et al., 2012; Hirata et al., 2015; Hubbard et al., 1993; Hunt et al., 1983; Mätz-Rensing et al., 1999; Paramastri et al., 2002; Suzuki et al., 2005). Thus, to the authors' knowledge, this is the first report on an IVL in a nonhuman primate.

In humans, clinical presentation and organ involvement of IVL are diverse. In patients from Western countries, the skin and central nervous system (CNS) are commonly affected with corresponding neurological symptoms (Glass et al., 1993; Ferreri et al., 2004; Ponzoni et al., 2007), whereas cases in the Asian population are typically characterized by bone marrow involvement and thrombocytopenia (Murase et al., 2000). Neurological symptoms, such as circling, head tilt, and nystagmus reflecting brain or spinal cord involvement, are among the most common clinical features of IVL in dogs (McDonough et al., 2002; Zuckerman et al., 2006; Lane et al., 2012). However, in the case reported herein, clinical signs were rather nonspecific with only subtle neurological symptoms including ataxia. The latter may have been a result of impaired cerebellar perfusion due to occlusion of cerebellar blood vessels by neoplastic lymphocytes (Lane et al., 2012) and/or of circulatory disturbance due to poor general condition.

Laboratory findings are not specific but indicative for IVL (Ponzoni et al., 2007). The most consistent hematological abnormalities in both humans and animals include anaemia, thrombocytopenia, and leukopenia (McDonough et al., 2002; Ferreri et al., 2004; Henrich et al., 2007; Lane et al., 2012). While the former two abnormalities occurred in the present case, the patas monkey showed a marked leukocytosis instead of a leukopenia. This finding is in line with the observed lymphocytic hyperplasia of the bone marrow. Due to the diversity of clinical manifestation and the resulting lack of specific diagnostic parameters, final diagnosis of IVL is often not established until postmortem examination (McDonough et al., 2002; Ferreri et al., 2004; Zuckerman et al., 2006; Lane et al., 2012). This was also true for the present case.

Histopathologic characteristics of IVL in both humans and animals include intravascular accumulation of large pleomorphic neoplastic lymphocytes in a variety of organs with common involvement of the CNS. Infiltration of adjacent parenchyma by neoplastic cells beyond endothelial lining is observed infrequently (McDonough et al., 2002; Ferreri et al., 2004; Henrich et al., 2007). The reason for this almost exclusive intravascular proliferation is poorly understood. In human B-cell IVL, deficiencies of the lymphoma cells in β-integrins and adhesion molecules (e.g., CD11a/CD18, CD29, CD54), which play a crucial role in lymphocyte extravasation, have been demonstrated and could be an explanation for the striking restriction to blood vessel lumina (Jalkanen et al., 1989; Ponzoni et al., 2000). Common secondary lesions of IVL are hemorrhage, oedema, and necrosis due to occlusion of vessels (McDonough et al., 2002; Ferreri et al., 2004; Henrich et al., 2007). The aforementioned histologic hallmarks are consistent with the findings in the present case, supporting the diagnosis of an IVL. Extensive secondary lesions were limited to the CNS. However, the macroscopically detectable hemorrhages in several organs apart from the CNS were considered to be most likely due to the severe thrombocytopenia, which probably led to hemorrhagic diathesis. Clinical studies have revealed that bone marrow involvement with intrasinusoidal localization of neoplastic lymphocytes occurs more frequently than previously anticipated (Estalilla et al., 1999; Dufau et al., 2000; Ferreri et al., 2004; Chaukiyal et al., 2006; Murase et al., 2007; Lane et al., 2012). In the patas monkey, careful histologic assessment revealed the presence of few neoplastic lymphoblasts, highlighted by CD3 expression, within bone marrow sinusoids as well. The follicular lymphoid hyperplasia, however, was characterized by small mature lymphocytes and hence was interpreted as a non-neoplastic reactive process. This assumption was supported by the predominance of T-cells, which were lined by fewer B-cells, as seen in benign lymphoid aggregates (Naemi et al., 2013).

The T-cell origin of the neoplastic cells in the present case, as demonstrated by CD3+ immunohistochemistry, is in line with the T-cell lineage predominant in domestic animals (McDonough et al., 2002). In contrast, 91 % of IVL in people is of B-cell phenotype (Estalilla et al., 1999; Ferreri et al., 2004), while only few reports exist on NK-cell and T-cell variants (Shimokawa et al., 1991; Wu et al., 2005; Cerroni et al., 2008). A further discrimination of neoplastic T-cells was not possible since neither neoplastic nor non-neoplastic lymphoid cells showed a reaction against anti-CD4 and anti-CD8 antibodies, suggesting a lack of cross-reactivity of the respective antibodies and the patas monkey epitopes. The strong nuclear Ki-67 signal indicates a high proliferative activity of the intravascular neoplastic cell population (Scholzen and Gerdes, 2000).

In light of the clear association between T-cell IVL and EBV infection in people (Au et al., 1997; Cerroni et al., 2008) and a suspected connection of simian LCVs and non-Hodgkin's lymphomas in macaques (Pingel et al., 1997; Mätz-Rensing et al., 1999; Blaschke et al., 2001; Hirata et al., 2015; Kahnt et al., 2002; Rivailler et al., 2004; Carville and Mansfield, 2008), the molecular detection of LCV made us initially suspect a gammaherpesviral-induced lymphomagenesis in the present case. However, since we were not able to demonstrate LCV immunohistochemically within neoplastic cells, the role of the LCV in oncogenesis remains elusive, in particular because of the high prevalence of the virus in nonhuman primates (Bruce et al., 2012). Moreover, infections with retroviral agents known to occur in patas monkeys such as STLV and SIV (Bibollet-Ruche et al., 1996; Nerrienet et al., 2001) and with the potential to induce lymphoid hyperplasia, immunodeficiency, and lymphomas (Lerche and Osborn, 2003) could not be ruled out with certainty since a serum sample of the patas monkey was not available. However, SIV and STLV were not detected by means of PCR in tissue specimens of the monkey.

In conclusion, clinical and pathomorphological features of the reported case of IVL in a patas monkey are consistent with the characteristics of the entity in humans and domestic animals. This case is the first description of this rare neoplasia in a nonhuman primate and again illustrates the difficulty of ante mortem diagnosis of IVL. A virus-induced lymphomagenesis was initially suspected but could not be verified in the present case.

## 5 Data availability

Underlying research data (histological slides) can be accessed upon request.

## References

[bib1.bib1] Allan JS, Leland M, Broussard S, Mone J, Hubbard G (2001). Simian T-cell lymphotropic viruses (STLVs) and lymphomas in African nonhuman primates. Cancer Invest.

[bib1.bib2] Au WY, Shek WH, Nicholls J, Tse KM, Todd D, Kwong YL (1997). T-cell intravascular lymphomatosis (angiotropic large cell lymphoma): Association with Epstein-Barr viral infection. Histopathology.

[bib1.bib3] Bibollet-Ruche F, Galat-Luong A, Cuny G, Sarni-Manchado P, Galat G, Durand JP, Pourrut X, Veas F (1996). Simian immunodeficiency virus infection in a patas monkey (Erythrocebus patas): evidence for cross-species transmission from African green monkeys (Cercopithecus aethiops sabaeus) in the wild. J Gen Virol.

[bib1.bib4] Blaschke S, Hannig H, Buske C, Kaup FJ, Hunsmann G, Bodemer W (2001). Expression of the simian Epstein-Barr virus-encoded latent membrane protein-1 in malignant lymphomas of SIV-infected rhesus macaques. J Med Virol.

[bib1.bib5] Bruce AG, Bielefeldt-Ohmann H, Barcy S, Bakke AM, Lewis P, Tsai CC, Murnane RD, Rose TM (2012). Macaque homologs of EBV and KSHV show uniquely different associations with simian AIDS-related lymphomas. PLoS Pathog.

[bib1.bib6] Bush WW, Throop JL, McManus PM, Kapatkin AS, Vite CH, van Winkle TJ (2003). Intravascular lymphoma involving the central and peripheral nervous systems in a dog. J Am Anim Hosp Assoc.

[bib1.bib7] Carville A, Mansfield KG (2008). Comparative pathobiology of macaque lymphocryptoviruses. Comp Med.

[bib1.bib8] Cerroni L, Massone C, Kutzner H, Mentzel T, Umbert P, Kerl H (2008). Intravascular large T-cell or NK-cell lymphoma: A rare variant of intravascular large cell lymphoma with frequent cytotoxic phenotype and association with Epstein-Barr virus infection. Am J Surg Pathol.

[bib1.bib9] Chaukiyal P, Singh S, Woodlock T, Dolan JG, Bruner K (2006). Intravascular large B-cell lymphoma with multisystem involvement. Leuk Lymphoma.

[bib1.bib10] Chmielewicz B, Goltz M, Lahrmann KH, Ehlers B (2003). Approaching virus safety in xenotransplantation: a search for unrecognized herpesviruses in pigs. Xenotransplantation.

[bib1.bib11] Clewley JP, Lewis JCM, Brown DWG, Gadsby EL (1998). A novel simian immunodeficiency virus (SIVdrl) *pol* Sequence from the Drill monkey, Mandrillus leucophaeus. J Virol.

[bib1.bib12] Cullen CL, Caswell JL, Grahn BH (2000). Intravascular lymphoma presenting as bilateral panophthalmitis and retinal detachment in a dog. J Am Anim Hosp Assoc.

[bib1.bib13] Dufau JP, Le Tourneau A, Molina T, Le Houcq M, Claessens YE, Rio B, Delmer A, Diebold J (2000). Intravascular large B-cell lymphoma with bone marrow involvement at presentation and haemophagocytic syndrome: Two Western cases in favour of a specific variant. Histopathology.

[bib1.bib14] Ehlers B, Ochs A, Leendertz F, Goltz M, Boesch C, Mätz-Rensing K (2003). Novel simian homologues of Epstein-Barr virus. J Virol.

[bib1.bib15] Estalilla OC, Koo CH, Brynes RK, Medeiros LJ (1999). Intravascular large B-cell lymphoma. A report of five cases initially diagnosed by bone marrow biopsy. Am J Clin Pathol.

[bib1.bib16] Ferreri AJ, Campo E, Seymour JF, Willemze R, Ilariucci F, Ambrosetti A, Zucca E, Rossi G, Lopez-Guillermo A, Pavlovsky MA, Geerts ML, Candoni A, Lestani M, Asioli S, Milani M, Piris MA, Pileri S, Fachetti F, Cavalli F, Ponzoni M (2004). Intravascular lymphoma: Clinical presentation, natural history, management and prognostic factors in a series of 38 cases, with special emphasis on the “cutaneous variant”. Br J Haematol.

[bib1.bib17] Ferry JA, Harris NL, Picker LJ, Weinberg DS, Rosales RK, Tapia J, Richardson Jr EP (1988). Intravascular lymphomatosis (malignant angioendotheliomatosis), A B-cell neoplasm expressing surface homing receptors. Mod Pathol.

[bib1.bib18] Glass J, Hochberg FH, Miller DC (1993). Intravascular lymphomatosis. A systemic disease with neurologic manifestations. Cancer.

[bib1.bib19] Habis A, Baskin GB, Murphey-Corb M, Levy LS (1999). Simian AIDS-associated lymphoma in rhesus and cynomolgus monkeys recapitulates the primary pathobiological features of AIDS-associated non-Hodgkin's lymphoma. AIDS Res Hum Retroviruses.

[bib1.bib20] Habis A, Baskin G, Simpson L, Fortgang I, Murphey-Corb M, Levy LS (2000). Rhesus lymphocryptovirus infection during the progression of SAIDS and SAIDS-associated lymphoma in the rhesus macaque. AIDS Res Hum Retroviruses.

[bib1.bib21] Henrich M, Huisinga M, Bauer N, Reinacher M (2007). A case of intravascular lymphoma with mixed lineage antigen expression in a cat. J Vet Med A.

[bib1.bib22] Hirata A, Hashimoto K, Katoh Y, Sakai H, Bruce AG, Rose TM, Kaneko A, Suzuki J, Nikami H, Yanai T (2015). Characterization of spontaneous malignant lymphomas in Japanese macaques (Macaca fuscata). Vet Pathol.

[bib1.bib23] Homma T, Kanki PJ, King Jr NW, Hunt RD, O'Connell MJ, Letvin NL, Daniel MD, Desrosiers RC, Yang CS, Essex M (1984). Lymphoma in macaques: association with virus of human T lymphotropic family. Science.

[bib1.bib24] Hubbard GB, Mone JP, Allan JS, Davis III KJ, Leland MM, Banks PM, Smir B (1993). Spontaneously generated non-Hodgkin's lymphoma in twenty-seven simian T-cell leukemia virus type 1 antibody-positive baboons (Papio species). Lab Anim Sci.

[bib1.bib25] Hunt RD, Blake BJ, Chalifoux LV, Sehgal PK, King NW, Letvin NL (1983). Transmission of naturally occuring lymphomas in macaque monkeys. P Natl Acad Sci USA.

[bib1.bib26] Jalkanen S, Aho R, Kallajoki M, Ekfors T, Nortamo P, Gahmberg C, Duijvestijn A, Kalimo H (1989). Lymphocyte homing receptors and adhesion molecules in intravascular malignant lymphomatosis. Int J Cancer.

[bib1.bib27] Kahnt K, Mätz-Rensing K, Hofmann P, Stahl-Hennig C, Kaup FJ (2002). SIV-associated lymphomas in rhesus monkeys (Macaca mulatta) in comparison with HIV-associated lymphomas. Vet Pathol.

[bib1.bib28] Lane LV, Allison RW, Rizzi TR, Stern AW, Snider TA, Moore PF, Vernau W (2012). Canine intravascular lymphoma with overt leukemia. Vet Clin Pathol.

[bib1.bib29] Lapointe JM, Higgins RJ, Kortz GD, Bailey CS, Moore PF (1997). Intravascular malignant T-cell lymphoma (malignant angioendotheliomatosis) in a cat. Vet Pathol.

[bib1.bib30] Leendertz SAJ, Junglen S, Hedemann C, Goffe A, Calvignac S, Boesch C, Leendertz FH (2010). High prevalences, coinfection rate, and genetic diversity of retroviruses in wild Red Colobus monkeys (Piliocolobus badius badius) in Tai National Park, Côte d'Ivoire. J Virol.

[bib1.bib31] Lerche NW, Osborn KG (2003). Simian retrovirus infections: potential confounding variables in primate toxicology studies. Toxicol Pathol.

[bib1.bib32] Li SL, Feichtinger H, Kaaya E, Migliorini P, Putkonen P, Biberfeld G, Middeldorp JM, Biberfeld P, Ernberg I (1993). Expression of Epstein-Barr-virus-related nuclear antigens and B-cell markers in lymphomas of SIV-immunosuppressed monkeys. Int J Cancer.

[bib1.bib33] Mätz-Rensing K, Pingel S, Hannig H, Bodemer W, Hunsmann G, Kuhn EM, Tiemann M, Kaup FJ (1999). Morphologic and immunophenotypic characteristics of malignant lymphomas in SIV-infected rhesus macaques. J Med Primatol.

[bib1.bib34] McDonough SP, Van Winkle TJ, Valentine BA, vanGessel YA, Summers BA (2002). Clinicopathological and immunophenotypical features of canine intravascular lymphoma (malignant angioendotheliomatosis). J Comp Pathol.

[bib1.bib35] Miller AD, Abee CR, Mansfield K, Tardif S, Morris T (2012). Neoplasia and proliferative disorders of nonhuman primates. Nonhuman primates in biomedical research.

[bib1.bib36] Murase T, Nakamura S, Kawauchi K, Matsuzaki H, Sakai C, Inaba T, Nasu K, Tashiro K, Suchi T, Saito H (2000). An Asian variant of intravascular large B-cell lymphoma: Clinical, pathological and cytogenetic approaches to diffuse large B-cell lymphoma associated with haemophagocytic syndrome. Br J Haematol.

[bib1.bib37] Murase T, Yamaguchi M, Suzuki R, Okamoto M, Sato Y, Tamaru J, Kojima M, Miura I, Mori N, Yoshino T, Nakamura S (2007). Intravascular large B-cell lymphoma (IVLBCL): A clinicopathologic study of 96 cases with special reference to the immunophenotypic heterogeneity of CD5. Blood.

[bib1.bib38] Naemi K, Brynes RK, Reisian N, Johnston A, Dhillon R, Walavalkar V, Zhao X, Rezk SA (2013). Benign lymphoid aggregates in the bone marrow: distribution patterns of B and T lymphocytes. Hum Pathol.

[bib1.bib39] Nakamura S, Ponzoni M, Campo E, Swerdlow SH, Campo E, Harris NL, Jaffe ES, Pileri SA, Stein H, Thiele J, Vardiman JW (2008). Intravascular large B-cell lymphoma. WHO classification of tumours of haematopoietic and lymphoid tissues.

[bib1.bib40] Nerrienet E, Meertens L, Kfutwah A, Foupouapouognigni Y, Gessain A (2001). Molecular epidemiology of simian T-lymphotropic virus (STLV) in wild-caught monkeys and apes from Cameroon: a new STLV-1, related to human T-lymphotropic virus subtype F, in a cercocebus agilis. J Gen Virol.

[bib1.bib41] Orzechowska BU, Powers MF, Sprague J, Li H, Yen B, Searles RP, Axthelm MK, Wong SW (2008). Rhesus macaque rhadinovirus-associated non-Hodgkin lymphoma: animal model for KSHV-associated malignancies. Blood.

[bib1.bib42] Paramastri YA, Wallace JM, Salleng KJ, Wilkinson LM, Malarkey DE, Cline JM (2002). Intracranial lymhomas in simian retrovirus-positive Macaca fascicularis. Vet Pathol.

[bib1.bib43] Pfleger L, Tappeiner J (1959). On the recognition of systematized endotheliomatosis of the cutaneous blood vessels reticuloendotheliosis. Hautarzt.

[bib1.bib44] Pingel S, Hannig H, Mätz-Rensing K, Kaup FJ, Hunsmann G, Bodemer W (1997). Detection of Epstein-Barr virus small RNAs EBER1 and EBER2 in lymphomas of SIV-infected rhesus monkeys by in situ hybridization. Int J Cancer.

[bib1.bib45] Ponzoni M, Arrigoni G, Gould VE, Del Curto B, Maggioni M, Scapinello A, Paolino S, Cassisa A, Patriarca C (2000). Lack of CD29 (beta1 integrin) and CD54 (ICAM-1) adhesion molecules in intravascular lymphomatosis. Hum Pathol.

[bib1.bib46] Ponzoni M, Ferreri AJ (2006). Intravascular lymphoma: A neoplasm of “homeless” lymphocytes?. Hematol Oncol.

[bib1.bib47] Ponzoni M, Ferreri AJ, Campo E, Facchetti F, Mazzucchelli L, Yoshino T, Murase T, Pileri SA, Doglioni C, Zucca E, Cavalli F, Nakamura S (2007). Definition, diagnosis, and management of intravascular large B-cell lymphoma: Proposals and perspectives from an international consensus meeting. J Clin Oncol.

[bib1.bib48] Raidal SL, Clark P, Raidal SR (2006). Angiotrophic T-cell lymphoma as a cause of regenerative anemia in a horse. J Vet Intern Med.

[bib1.bib49] Rivailler P, Carville A, Kaur A, Rao P, Quink C, Kutok JL, Westmoreland S, Klumpp S, Simon M, Aster JC, Wand F (2004). Experimental rhesus lymphocryptovirus infection in immunosuppressed macaques: An animal model for Epstein-Barr virus pathogenesis in the immunosuppressed host. Blood.

[bib1.bib50] Scholzen T, Gerdes J (2000). The Ki-67 protein: from the known to the unknown. J Cell Physiol.

[bib1.bib51] Sheibani K, Battifora H, Winberg CD, Burke JS, Ben-Ezra J, Ellinger GM, Quigley NJ, Fernandez BB, Morrow D, Rappaport H (1986). Further evidence that “malignant angioendotheliomatosis” is an angiotropic large-cell lymphoma. N Engl J Med.

[bib1.bib52] Shimokawa I, Higami Y, Sakai H, Moriuchi Y, Murase K, Ikeda T (1991). Intravascular malignant lymphomatosis: A case of T-cell lymphoma probably associated with human T-cell lymphotropic virus. Hum Pathol.

[bib1.bib53] Sly DL, London WT, Palmer AE, Rice JM (1978). Growth and hematologic development of the patas monkey (*Erythrocebus patas*) to one year of age. J Med Primatol.

[bib1.bib54] Suzuki J, Goto S, Kato A, Hashimoto C, Miwa N, Takao S, Ishida T, Fukuoka A, Nakayama H, Doi K, Isowa K (2005). Malignant NK/T-cell lymphoma associated with simian Epstein-Barr virus infection in a Japanese macaque (Macaca fuscata). Exp Anim.

[bib1.bib55] Wick MR, Mills SE (1991). Intravascular lymphomatosis: Clinicopathologic features and differential diagnosis. Semin Diagn Pathol.

[bib1.bib56] Wick MR, Mills SE, Scheithauer BW, Cooper PH, Davitz MA, Parkinson K (1986). Reassessment of malignant “angioendotheliomatosis” Evidence in favor of its reclassification as “intravascular lymphomatosis”. Am J Surg Pathol.

[bib1.bib57] Wu H, Said JW, Ames ED, Chen C, McWhorter V, Chen P, Ghali V, Pinkus GS (2005). First reported cases of intravascular large cell lymphoma of the NK cell type: Clinical, histologic, immunophenotypic, and molecular features. Am J Clin Pathol.

[bib1.bib58] Zuckerman D, Seliem R, Hochberg E (2006). Intravascular lymphoma: The oncologist's “great imitator”. Oncologist.

